# A natural interomone 2-methyl-2-butenal stimulates feed intake and weight gain in weaned pigs

**DOI:** 10.1017/S1751731116001439

**Published:** 2016-07-13

**Authors:** J. J. McGlone, G. Thompson, S. Devaraj

**Affiliations:** 1 Animal and Food Sciences Department, Laboratory of Animal Behavior, Physiology and Welfare, Texas Tech University, Lubbock, TX 79409-2141, USA; 2 Animal Biotech, LLC., Dallas, TX 75201, USA

**Keywords:** interomone, rabbit pheromone, pigs, weight gain

## Abstract

A novel approach to potentially improve pig growth and welfare is to supplement environments with biologically meaningful odors that are deficient. The post-weaning environment lacks maternal odors that may contribute to the often-observed post-weaning lag in growth and health challenges. A recently reported rabbit maternal pheromone (2-methyl-2-butenal (2M2B)) may act as an interomone in the pig. The objective of this study was to determine if providing a maternal pheromone/interomone during transport and the post-weaning environment may enhance pig performance. A total of 40 replicated pens were used in a factorial arrangement of two transport olfactory experiences (Control *v*. 2M2B), two nursery olfactory experiences (Control *v*. 2M2B) and two sexes (barrow *v*. gilt). Pig body weight, average daily gain (ADG), average daily feed intake (ADFI) and gain : feed ratio (G : F) were measured and calculated over a 28-day post-weaning period. Pig sex and application of 2M2B during transport had no effect on pig performance. However, pigs that had 2M2B applied to their feeder at weaning had 15% greater feed intake (0.74 *v*. 0.64±0.03 kg/day, *P*<0.01) and 12% greater ADG (0.27 *v*. 0.24 kg/day, *P*<0.05) than control pigs. G : F ratio was not different between treatments. The interomone 2M2B is a novel, safe molecule that can improve pig post-weaning performance. This report highlights a new area of study and a natural class of compounds that can improve pig performance and potentially improve pig welfare.

## Implications

Separation of the piglet from the sow at weaning is stressful to the piglet. When a natural, safe flavor or sow odor (2-methyl-2-butenal (2M2B)) was placed in the post-weaning environment, piglets ate more feed and gained more weight than control piglets who did not have this flavor or odor. Use of natural flavors or odors may improve weaned piglet growth and health and may provide a positive animal welfare tool.

## Introduction

Piglets are commonly weaned about 21 days of age. Weaning presents a stress in that piglets experience changes in diet, and physical and thermal environments. Importantly, weaned piglets also experience a change in social and olfactory environments that contribute to the stress of weaning. Weaning causes weight loss, increased blood cortisol concentrations, a depressed immune system and significant behavioral changes (Blecha *et al*., 1983). Antibiotics are commonly used in weaned pigs to reduce the negative effects of weaning. However, social concerns about the use of antibiotics have led to a search for natural alternatives. One view of the post-weaning environment is that it lacks in familiar, comforting semiochemicals including pheromones. At the same time, pheromones can be used to modify behavior of pigs because pigs rely heavily on olfactory signals. Applying semiochemicals or a related class of chemicals to the post-weaning environment may reduce the deficiency of maternal and other behavior-modulating chemical signals.

The boar pheromone (androstenone) reduced pig aggressive behaviors and can temporarily improve weight gain of re-grouped, stressed pigs (McGlone *et al*., 1986; McGlone and Morrow, 1988). A putative maternal pheromone that is a mixture of fatty acids found on the mammary surfaces of lactating sows also was shown to increase post-weaning weight gain (McGlone and Anderson, 2002). Another class of semiochemicals are the interomones. An interomone is a pheromone in one species that changes the behavior or physiology of another species without the requirements to benefit or harm, the emitter or the receiver (McGlone, 2014). Here we examined the role of the rabbit nipple-searching pheromone (2M2B; Schaal *et al*., 2003), which possibly acts as a flavor or interomone in the post-weaning environment on weaned pig performance.

## Material and methods

All studies involving animals were approved by the Institutional Care and Use Committee before the conduct of the work. All procedures were consistent with the Guide for the Care and Use of Agricultural Animals (McGlone *et al*., 2010). None of the studies involved pain or distress. The parents of the subject animals were Pig Improvement Company (PIC) USA Camborough genetics. The work was conducted in laboratories of Texas Tech University. All animal assessments were performed in commercial-type farm facilities. Pigs were fed a corn–soybean meal diet formulated to meet or exceed the NRC (2012) nutrient requirements of swine.

Pigs (*n*=120) were randomly selected from nursing sows. A total of 60 males and 60 females were weaned at ~3 weeks of age and transported for 4 h before entering the nursery site. Pigs were housed in groups of three single-sex (either barrows or gilts) and had *ad libitum* access to feed and water. A total of 40 pens (the experimental unit) were available; 10 each of male and female experiencing control or pheromone therapy. Space allowance was in excess of industry standards (0.6 m^2^/pig). Body weight and feed intake were measured once per week from weaning until 28 days after weaning.

Control and 2M2B-treated pigs were housed in separate rooms to ensure no cross-contamination between air spaces. The control feeders received 25 ml of isopropyl alcohol while the treated feeders received 25 ml of the trans-2-methyl-2-butenal (also called Tiglic aldehyde; CAS 497-03-0; Sigma-Aldrich, St. Louis, MO, USA) solution (1 µg/ml in isopropyl alcohol). Feeders were sprayed immediately before the arrival of piglets to the nursery site. Half of the pigs and pens in each group were transported and half were moved directly from the farrowing crates to the adjacent nursery building. The experimental design was a completely random design with a two by two factorial arrangement of transport (or not) and 2M2B or placebo control. Average daily gain (ADG, kg/day) was calculated from the BWs both weekly and over the entire period. Average daily feed intake (ADFI kg/pig per day) was calculated based on weekly feed consumption. Gain : Feed ratio (G : F) was calculated each week and over the 28-day post-weaning period.

Data were analyzed using SAS software (SAS, 2012). PROC Univariate was used to establish that the overall ADG, ADFI and G : F ratio data were normally distributed. The general linear models procedure was used with pen as the experimental unit. There were 40 experimental units (pens) in total. The original model contained the factorial effects of (A) 2M2B applied during transport or not, (B) 2M2B applied to the nursery feeder or not, and (C) pig sex (barrow *v*. gilt) and all possible interactions. For overall performance measures, the effects of transport, application of 2M2B, sex and the interaction of these variables with 2M2B applied in the nursery were not significant (*P*>0.10). A final simplified analysis was performed with only the effects of 2M2B applied in the nursery or not with 20 pens per treatment.

## Results

For all performance measures, the interaction between transport and 2M2B application was not significant (*P*>0.10). Transport for 4 h at the time of weaning did not have any long-term effect on pig performance. Sex effects and the sex by treatment interactions were not significant, except for BW data in week 1. In other week and over the entire period, the sex effects and sex effect interactions with other treatments were not significant. Table 1 shows the results of pig performance measures. Pig BW were statistically similar at weaning and 7 days later. At 14 days after weaning, pigs experiencing 2M2B tended (*P*=0.06) to be heavier. On day 21 after weaning, pigs exposed to 2M2B were heavier (*P*<0.05) than control pigs.

**Table 1:** Post-weaning pig performance least squares means

Item	Control	2M2B	SE	*P*-value
BW (kg)				
Weaning (3 weeks of age)	6.5	6.6	0.06	0.70
7 days	7.1	7.3	0.11	0.20
14 days	8.1	8.4	0.13	0.06
21 days	10.1	10.9	0.18	0.003
28 days	13.3	14.1	0.25	0.02
ADG (kg/day)				
Week 1	0.08	0.11	0.01	0.16
Week 2	0.15	0.16	0.01	0.23
Week 3	0.28	0.35	0.02	0.005
Week 4	0.46	0.46	0.02	0.83
ADFI (kg/pig per day)				
Week 1	0.15	0.17	0.01	0.25
Week 2	0.27	0.30	0.02	0.18
Week 3	0.47	0.53	0.02	0.055
Week 4	0.64	0.74	0.03	0.009
G : F				
Week 1	0.36	0.59	0.20	0.44
Week 2	0.57	0.55	0.03	0.83
Week 3	0.59	0.67	0.02	0.10
Week 4	0.76	0.62	0.05	0.07
Overall performance				
ADG (kg/day)	0.24	0.27	0.01	0.027
ADFI (kg/day)	0.38	0.44	0.02	0.007
G : F ratio	0.63	0.62	0.07	0.56

ADG=average daily gain; ADFI=average daily feed intake; G : F=gain : feed ratio; 2M2B=2-methyl-2-butenal. *n*=20 pens per treatment group. The *P*-value refers to the significance of the control v. the 2M2B treatments.

Application of 2M2B caused an increase (*P*<0.05) in overall ADG and ADFI during 4 weeks after weaning. The G : F ratio was not influenced (*P*>0.10) by treatments. Application of 2M2B increased post-weaning ADG in week 3 and overall (*P*<0.05). ADFI was higher (*P*<0.05) among 2M2B-treated pens in week 3 and 4 after weaning and during the overall growth period. G : F ratio was not significantly changed by 2M2B. The effect of 2M2B was that it stimulated feed intake and BW gain. At weaning, control pigs weighed 6.5±0.06 kg and pigs to experience 2M2B weighed 6.6±0.02 kg (*P*>0.10). Four weeks later, control pigs weighed 13.3±0.25 kg while pigs experiencing 2M2B weighed 14.1±0.25 kg, a 6% improvement (*P*=0.01).

## Discussion

The post-weaning environment is deficient in familiar and perhaps comforting maternal odors. We hypothesize that part of the post-weaning lag in growth and challenges in pig health is due to a sudden loss of maternal odors that are meaningful to the pig. Tiglic aldehyde, or 2M2B is naturally found in berries, some vegetables, chicken fat and of course in mammary secretions of rabbits (Schaal *et al*., 2003). It is sold as a food-grade flavoring agent and generally recognized as safe by the flavor and extract manufacturers association of the United States (Flavor and Extract Manufactures Association (FEMA), 2016). In the rabbit pups, 2M2B induces nipple search behavior (Schaal *et al*., 2003). We have examined the role of 2M2B in weaned pig performance. To be called a pheromone, 2M2B would have be found to be in any secretion of a pig and to have an effect on the physiology or behavior of another pig (Karlson and Luscher, 1959). A previous report found 2M2B in the cooked pork liver (Mussinan and Walradt, 1974), however, it is not a known pheromone in the pig. At this time, 2M2B has not yet been shown to be present in pig secretions or to impact pig physiology or behavior.

Semiochemicals or volatile compounds are conserved over plant and animal species (like 2M2B) and a given molecule may serve different functions. For example, the bark beetle aggregation pheromone is a sex pheromone in elephants (Rasmussen *et al*., 2016). Different species cannot easily use the same pheromone for the same purpose in the same ecosystem or there would be biological confusion. However, the same molecule may be used for different species for different purposes without invoking biological confusion. Different species are likely to have olfactory receptors for volatile compounds that operate as a pheromone in a given species. With this logic, we began searching for interomone effects among known pheromones and we understand that the rabbit maternal–neonatal pheromone has effects on pigs.

Interomones are an interesting class of compounds because they are natural, powerful at low concentrations and generally safe in part because they are sprayed on the environment and not injected or fed (although doing so might also have interested effects). The interomone of the present study, 2M2B was sprayed on the feeder in part in an attempt to find an alternative to administering medically important antibiotics as growth promotants. This interomone does not act as a drug, but is a new class of natural compounds more akin to a nutrient of sorts. We hypothesize that the livestock production environments (and other animal environments like companion animal homes and zoos) do not provide for all of the olfactory ‘needs’ of animals. By supplementing the olfactory environment with deficient semiochemicals, odors or flavors we may be able to improve animal performance and welfare.

Clearly, 2M2B, when added in a single application to the feeder in the post-weaning environment, stimulated ADFI and ADG in piglets. It could be that 2M2B stimulates feeding behavior, feed intake and increased growth by acting as an interomone or food-flavoring agent. The stimulatory effect on feed intake is very small (and not significant) during the 1st week after weaning, but it increases in magnitude in week 2 to 4 after weaning. During the period of increased growth, the 2M2B that was applied is likely to not be still present, as it is volatile. We are exploring mechanisms of action of 2M2B at this time and conducting studies in different farms with large number of animals to validate the performance difference among the pigs. It is also likely that 2M2B would have positive effects on other species especially during times in which maternal semiochemicals are abruptly removed, but also, perhaps, during other times of stress. Moreover, we have unpublished data showing that 2M2B has positive effects on physiology and behavior of cats and dogs. Therefore, 2M2B could have broad, positive effects on farm, companion and perhaps other animals.
